# Educational agents and institutions called into action in suicide prevention, intervention, and postvention

**DOI:** 10.3389/fpsyg.2023.1213751

**Published:** 2023-09-14

**Authors:** Janaina Minelli De Oliveira, Jorge-Manuel Dueñas, Fabia Morales-Vives, Elena Gallardo-Nieto

**Affiliations:** ^1^Pedagogy Department, Universitat Rovira i Virgili, Tarragona, Spain; ^2^Research Center for Behavior Assessment, Psychology Department, Universitat Rovira i Virgili, Tarragona, Spain; ^3^Universitat Ramón Llull, FPCEE, Blanquerna, Spain

**Keywords:** suicide prevention, suicide intervention, suicide postvention, health education, educational change, mental health, school mental health, student mental health

## Abstract

**Introduction:**

Suicide is the second leading cause of death in the 15 to 29 age group worldwide, and is a severe public health problem. Adolescent and young adult individuals attend educational institutions which can play an essential role in detecting and preventing suicide. For this reason, the purpose of this research is to identify what educational institutions and agents are called into action in suicide prevention, intervention, and postvention.

**Methods:**

The method of systematic review of the literature based on the PRISMA protocol was used. The review protocol was registered in PROSPERO (PROSPERO 2020 CRD42020189127). The systematic review yielded 66 articles published between 1990 and February 2023.

**Results:**

The results show that a wide variety of educational stakeholders are required to intervene for suicide prevention, interventions and postvention between primary education and college. The study describes the different programs that have been provided, the countries in which they have been implemented and the agents who have been targeted. It also identifies gaps in the research on suicide in the educational field.

**Discussion:**

Overall, educational suicide initiatives report positive effects on participants’ understanding, attitudes, and beliefs regarding suicide and suicide prevention, although some studies have expressed some caution.

## 1. Introduction

Suicide is a serious global public health issue (Cheng et al., 2020; World Health Organization, 2021a; Canbolat and Gençöz, 2023; Imran et al., 2023; Naveed et al., 2023). Every suicide is a tragedy that affects families, communities, and entire countries (Bengesser et al., 2000; World Health Organization, 2021b) and has long-lasting effects on the people left behind (Cain, 2002; Avrami, 2003; Mintz-Binder, 2007; Rosenberg, 2017; Vidal-Ribas et al., 2021; Connell et al., 2022). The reduction of suicide mortality has been prioritized by the World Health Organization (WHO) as a global target and included as an indicator in the United Nations Sustainable Development Goals (SDGs) under target 3.4, the WHO 13th General Program of Work 2019-20231 and the WHO Mental Health Action Plan 2013–2030. Suicide occurs throughout the lifespan and was the fourth leading cause of death among 15–29 year-old globally in 2019 (World Health Organization, 2021a).

A significant problem is preventing suicide in adolescents and young people (Greydanus et al., 2009; Sood and Linker, 2017; Sherman and Torga, 2022; Williams et al., 2022). The World Health Organization considers the educational environment excellent for suicide prevention (World Health Organization, 2020). The research indicates that there is a great need to address suicide-related mental health problems in schools (Brown and Grumet, 2009; Hooven et al., 2012; Singer, 2017; Mirick and Berkowitz, 2022). One of the saddest aspects of teen suicide is the frequently missed opportunity to stop it (Mulrine, 2001). Many studies underscore the importance of suicide prevention education throughout the high school and college years (King et al., 2008; Joshi et al., 2015; Poland and Ferguson, 2021; Testoni et al., 2021; Chaniang et al., 2022; Stickl Haugen et al., 2022; Fadillah et al., 2023). However, difficulties associated to staff shortages, ever-increasing responsibilities for student well-being (Ayer et al., 2022) and shortage of guidelines on the targets and methods of safe and effective awareness programs are highlighted (Grosselli et al., 2022). In the context of education, little study has precisely outlined who should be in charge of what actions. Schools and universities can and should play a big role in fostering discussion with young people about the subject, but more needs to be done (Burlea et al., 2012; deCou et al., 2019; Malhi and Bell, 2020; Shand and Torok, 2022). The objective of the present paper is to identify what educational institutions and agents are called into action in suicide prevention, intervention, and postvention.

In pursuit of our research goals, a systematic review is justified by the relevance and seriousness of the suicide problem worldwide. It is imperative to exhaustively identify and analyze which institutions and educational agents are called upon to act in the prevention, intervention, and postvention of suicide, considering the vital role that educational institutions can play in the early detection and prevention of this tragic phenomenon. Systematic reviews are a rigorous research methodology that allows for an objective and comprehensive synthesis of the existing literature on a specific topic. In this case, the PRISMA protocol was used to ensure a systematic and transparent collection of relevant studies related to the role of educational institutions and agents in suicide prevention. It is essential to distinguish systematic reviews from scoping reviews. While scoping reviews map the existing literature and detect key topic areas, systematic reviews answer specific research questions by identifying, selecting, and synthesizing relevant studies that meet quality and validity criteria. The implications of this systematic review are significant for policy-making and practice in suicide prevention in educational institutions. The findings have important practical implications for educational professionals and staff.

Previous systematic reviews have done the effort to bring together suicide prevention, a topic more directly associated to health, and the educational field. For example, Katz et al. (2013) conducted a systematic review of the empirical literature on school-based suicide prevention programs. This interesting previous study covers a time span from 1966 to 2012, focusing on MEDLINE and Scopus databases. Systematic reviews of Mo et al. (2018) and Torok et al. (2019) focused on gatekeeper training programs. Systematic review of Li et al. (2019) focused on suicide risk in college students. This new review, which we present here, is necessary because it is the first review to identify educational agents called into action and recommendations made for the last three decades of research, even though we acknowledge that these earlier reviews made significant contributions to the open discussion of suicide prevention in the educational field. Furthermore, we classify the programs as presenting prevention, intervention, or postvention initiatives, also considering the educational setting addressed, from primary school to college. By identifying the different academic actors involved in suicide prevention, greater collaboration and coordination can be promoted to implement effective evidence-based interventions. In addition, possible research gaps can be identified, suggesting the need to direct future research toward specific areas that still need to be sufficiently explored.

## 2. Methods

This review follows a broadly aggregative synthesis logic (Hart, 1998) and is registered in PROSPERO International prospective register of systematic reviews (PROSPERO 2020 CRD42020189127). The body of evidence provided here shows systematically that existing primary research results contain arguments to shape and inform practice and policies (Zawacki-Richter et al., 2020).

The research team gathered the following specific research questions that embodied scientific motivation:

1. What educational agents and institutions have been identified in the literature as settings for suicide prevention and why?
2. What prevention, intervention, and postvention programs have been carried out in the educational setting to reduce suicidal manifestations?

Based on these research questions, the authors set the main objective of this study, which is to identify what educational institutions and agents are called into action in suicide prevention, intervention, and postvention. The study also aimed to inform both the research community and policymakers on how to address future research questions and revise educational policies on suicide prevention. Based on the existing research, our hypothesis was that the literature would identify the educational stakeholders who should play a role in suicide prevention, intervention, and postvention and offer recommendations that may guide educational stakeholders when approaching suicide prevention, intervention, and postvention in educational settings.

### 2.1. Data source and searching strategies

The search strategy was defined in discussions held by the authors. The search terms were determined based on keywords identified in preliminary searches. Boolean operators and search terms used were: TITLE: (suici* near/5 education) OR TITLE: (suici* near/5 school*) OR TITLE: (suici* near/5 university) OR TITLE: (suici* near/5 teacher*) OR TITLE: (suici* near/5 student*) OR TITLE: (suici* near/5 educator*). The search for each keyword and phrase was done in an individual search. The authors searched the WOS, CCC, DIIDW, KJD, MEDLINE, RSCI, and SCIELO databases. We examined articles published between 1990 and February 2023, thus covering more than three decades of research on educational initiatives of suicide prevention.

### 2.2. Eligibility criteria

The studies selected to be included in this review had to specifically relate to suicide prevention, intervention, or postvention in an educational setting. Moreover, they had to describe and/or assess an educational intervention specifically designed for suicide prevention, intervention, and postvention, to raise awareness of suicide-related themes, to identify and/or support at-risk groups, to promote protective factors for suicide, to offer first aid in a suicide-related emergency, and to address postvention. Finally, the studies had to have been published in a peer-reviewed journal between 1990 and 2023—data analysis finished on the 31st of February 2023. Only studies written in English were included.

Studies were excluded from the review if they did not specifically address educational aspects of suicide prevention, were not published in a peer-reviewed journal, or contained no unique relevant data about the inclusion criteria. Research trials and screenings which did not report on education intervention results were also excluded. Studies written in a language other than English were excluded.

### 2.3. Extraction and screening

The search strategy described above retrieved 1,294 items, which were downloaded to Endnote. After duplicate items were removed, 1,127 articles remained. The authors then conducted a pilot study in which they analyzed 10% of the corpus. After the pilot stage, we adjusted the eligibility criteria to exclude research trials and screenings that did not report on results of educational interventions, even when they addressed suicide prevention initiatives. The remaining article titles and abstracts retrieved were systematically screened by three of the co-authors in an initial process to select and remove items by applying the refined inclusion and exclusion criteria. The extraction of data from all relevant papers was completed at this point using an online Excel document shared by the authors. Research meetings were held to discuss questionable items. Ninety-four (94) articles were selected by two or three researchers to compose the corpus of the second stage of the analysis. This was reduced to a final corpus of (66) articles after the inclusion and exclusion criteria were revised, this time after reading the full manuscripts. The (66) papers in the final corpus were uploaded to the software Atlas.ti.

### 2.4. Quality analysis

Three people participated in the evaluation of the articles to determine their quality and risk of bias. More specifically, two reviewers independently undertook the quality assessment of the articles, and disagreements were resolved by discussion or by a third reviewer if necessary. Due to the variety of methodological approaches used by researchers to pursue their objectives, different assessment tools were used to assess the trustworthiness, relevance, and results of papers obtained. Downloadable checklists for Randomized Controlled Trials of Critical Appraisal Skills Programme (2022a,b,c), Cohort Studies and Qualitative Studies were used. We used JBI’s tools for assessing quasi experimental studies (Tufanaru et al., 2020) and text and opinion (McArthur et al., 2020). The Ways of Evaluating Important and Relevant Data (WEIRD) tool (Lewin et al., 2019) was also used. A study was considered to have an adequate methodological quality when it met at least 70% of the criteria specified in the assessment tool used. At this point, no articles were excluded.

### 2.5. Data coding

**Figure 1:**
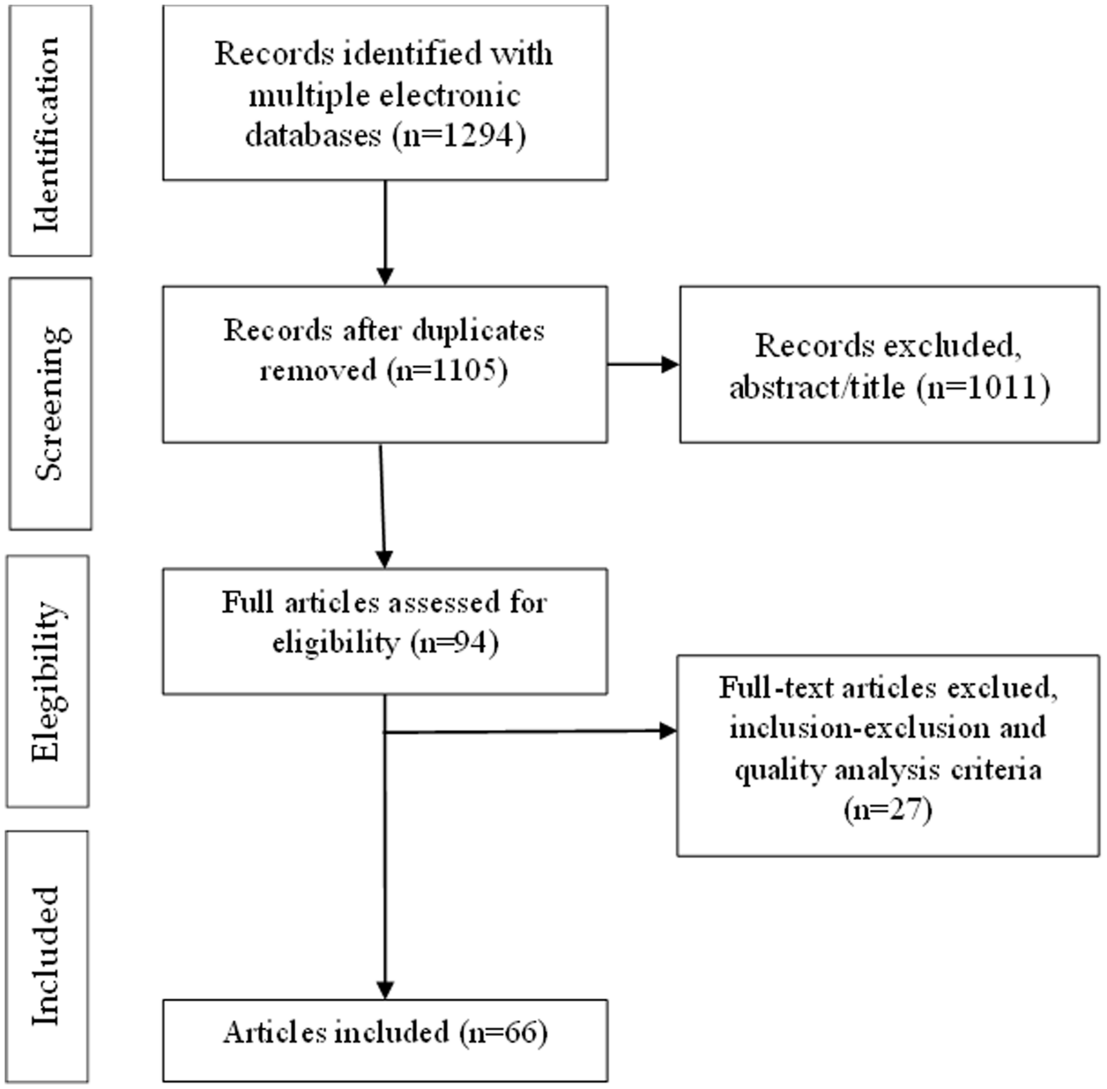
PRISMA flow chart of the selection procedure.

The (66) papers in the final corpus uploaded to the software Atlas.ti were coded for type of educational institution (e.g., school, university, and others), agents (e.g., teachers, school directors, parents, social educators, and policymakers), agents’ skills and knowledge (e.g., suicide risk and protective factors, and crisis management), research objectives pursued, recommendations for educational stakeholders, and future research directions suggested. Figure 1 shows the procedure for applying the PRISMA criteria (Moher et al., 2015).

## 3. Results

**Figure 2:**
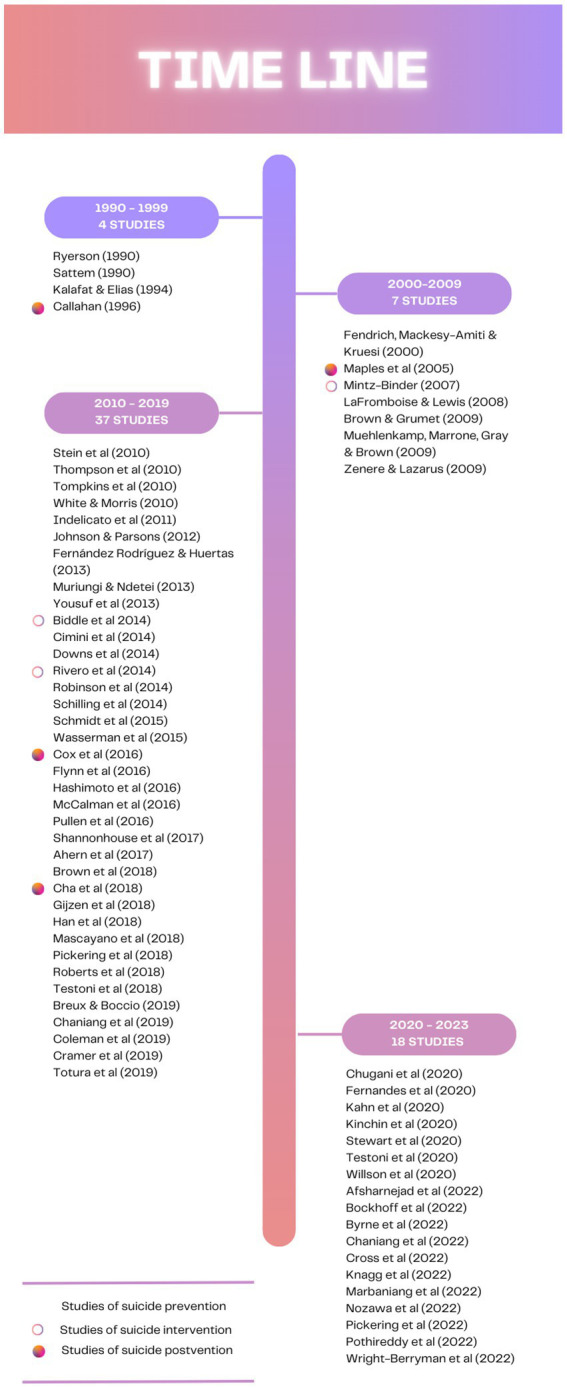
Timeline of studies describing and or assessing educational programs targeted at suicide prevention, intervention, or postvention published between 1990 and February 2023.

Sixty-six studies published between 1990 and February 2023 described and/or assessed an educational intervention on suicide prevention (59), intervention (3), or postvention (4). Figure 2 shows the different articles analyzed in this study across a timeline. As can be seen in the figure, most of the articles were published in the 2010–2019 decade, and there are few articles for the 1990–1999 and 2000–2009 decades. Regarding the 1990–1999 decade, three articles focused on prevention programs and one focused on a postvention program. In the 2000–2009 decade, one study focused on a postvention program, one focused on an intervention program and four focused on prevention programs. Likewise, in the 2010–2019 decade, all the studies but four focused on prevention programs. Two of them analyzed intervention programs and two analyzed postvention programs. Finally, all the studies published in the 2020–2022 years focused on prevention programs. Therefore, prevention studies predominate in each decade. The considerable number of studies published since 2010 suggests that there has been a growing interest for this area of research, although this interest still focuses primarily on prevention programs.

Table 1 summarizes the various methodological approaches used by researchers to pursue their objectives. Of the 66 studies, 50 (75.75%) used quantitative methods, 12 (18.18%) used qualitative methods, and two (3.03%) used a mixed-method approach. The methodology applied in two (3.03%) of the studies was not clearly described. Thirty-nine programs designed to approach suicide prevention (34), intervention (3), or postvention (2) in educational settings were described and/or assessed in the literature. Table 2 presents the list of programs identified, a brief description of the programs, and the study in which they appear. The educational settings addressed by the studies ranged from primary school to college. Table 3 classifies the studies in terms of the suicide phase and educational setting.

**Table 1:** Methodological approaches of the 66 studies published between 1990 and February 2023 reporting a description or assessment of suicide educational interventions.

Methodological approach	*N*	Studies
Randomized trial	14	Robinson et al. (2014); Wasserman et al. (2015); Ahern et al. (2018); Gijzen et al. (2018); Han et al. (2018); Mascayano et al. (2018); Pickering et al. (2018); Roberts et al. (2018); Coleman et al. (2019); Kahn et al. (2020); Afsharnejad et al. (2022); Byrne et al. (2022); Nozawa et al. (2022); Pickering et al. (2022)
Pre-post study design	15	Cramer et al. (2019); Flynn et al. (2016); Johnson and Parsons (2012); LaFromboise and Lewis (2008); Muehlenkamp et al. (2009); Pothireddy et al. (2022); Shannonhouse et al. (2017); Stewart et al. (2020); Testoni et al. (2018, 2020); Tompkins et al. (2010); Totura et al. (2019); Willson et al. (2020); Wright-Berryman et al. (2022); Yousuf et al. (2013)
Pre-post study designs with follow up	11	Indelicato et al. (2011); Cimini et al. (2014); Schilling et al. (2014); Hashimoto et al. (2016); Pullen et al. (2016); Brown et al. (2018); Cha et al. (2018); Breux and Boccio (2019); Kinchin et al. (2020); Knagg et al. (2022); Marbaniang et al. (2022)
Post-intervention study design	6	Ryerson (1990); Fendrich et al. (2000); Brown and Grumet (2009); Thompson et al. (2010); Downs et al. (2014); Cross et al. (2022)
Experimental study	1	Bockhoff et al. (2022)
Case study	4	Callahan (1996); Rivero et al. (2014); Schmidt et al. (2015); Chaniang et al. (2022)
Mixed-methods design, drawing on complementary quantitative and qualitative data	2	McCalman et al. (2016); Chaniang et al. (2019)
Qualitative study using key informant interviews or discussion groups	3	Stein et al. (2010); Fernández Rodríguez and Huertas (2013); Chugani et al. (2020)
Solomon four-group design	1	Kalafat and Elias (1994)
Clinical trial	1	Muriungi and Ndetei (2013)
Inferential, retrospective, secondary regression analysis	1	Biddle et al. (2014)
Longitudinal analysis	1	Zenere and Lazarus (2009)
In-depth qualitative case study, discursively oriented	1	White and Morris (2010)
Qualitative descriptive exploratory research	1	Fernandes et al. (2020)
First-person account	1	Maples et al. (2005)
Delphi methodology	1	Cox et al. (2016)
Not clearly described	2	Sattem (1990); Mintz-Binder (2007)

**Table 2:** Educational programs addressing prevention, intervention, or postvention described or assessed in studies published between 1990 and February 2023.

Program name	Brief description	Studies
	*Prevention*	
Puppet Prevention Program	A youth-system-based prevention and early identification process that uses puppets.	Sattem (1990)
Adolescent Suicide Awareness Program (ASAP)	A mental health education program for school communities designed to be implemented as a cooperative project between community mental health providers and local school systems.	Ryerson (1990)
Youth Suicide Prevention and Intervention Program	Universal suicide prevention strategies are implemented through the To Reach Ultimate Success Together curriculum in a series of skill-development lessons.	Zenere and Lazarus (2009)
STOP Suicide Program (School-Based Teen Outreach Program for Suicide)	A program funded by the Substance Abuse and Mental Health Services Administration housed in the DC Department of Mental Health, United States.	Brown and Grumet (2009)
The Medicine Wheel Program	A culturally informed circle-of-care approach that builds upon mainstream suicide prevention strategies by incorporating traditional American Indian (AI) practices, knowledge, and outreach.	Muehlenkamp et al. (2009)
Youth Suicide Prevention Program (YSPP)	The Los Angeles Unified School District’s (LAUSD) suicide prevention program.	Stein et al. (2010)
Question, Persuade, Refer (QPR)	Gatekeeper training in an educational setting to identify and intervene when individuals are engaged in risky behaviors.	Tompkins et al. (2010); Indelicato et al. (2011); Johnson and Parsons (2012); Fernández Rodríguez and Huertas (2013); Muriungi and Ndetei (2013);Wasserman et al. (2015); Pullen et al. (2016); Ahern et al. (2018); Willson et al. (2020)
Signs of Suicide (SOS)	A 17-min DVD that includes (1) three age-appropriate vignettes that are less intense than the high school version; (2) a group discussion by middle school students about depression, suicide, bullying, self-injury, and getting help; and (3) a student interview with a school-based counselor to model getting help.	Schilling et al. (2014)
Yellow Ribbon Suicide Prevention Program (YRSPP)	The program integrates education on help-seeking behaviors and screening.	Schmidt et al. (2015); Flynn et al. (2016)
Youth Aware of Mental Health program (YAM)	Promotes knowledge of mental health, healthy lifestyles, and behaviors.	Wasserman et al. (2015); Ahern et al. (2018); Kahn et al. (2020)
Aussie Optimism Program (AOP)	A prevention educational program was implemented as a community-based project in collaboration with school nurses.	Roberts et al. (2018)
Screening by Professionals (ProfScreen)	A two-stage screening tool to help health professionals to identify at-risk adolescents based on mental health responses in a self-report questionnaire.	Wasserman et al. (2015); Ahern et al. (2018)
Kognito At Risk (see www.kognito.com)	The trainee interacts with virtual peers and is given a menu of choices for interactions. They are led to identify peers who may be at risk.	Coleman et al. (2019)
Healer Education Assessment and Referral (HEAR) program	This program uses secondary and tertiary prevention strategies to address depression and suicide.	Downs et al. (2014)
Suicide Prevention Program (SPP)	The program involves a collaborative model that engages every sector of the university.	Fernández Rodríguez and Huertas (2013)
Multimodal stepped-prevention program	The program comprises screening with subsequent clinical evaluation and/or referral; gatekeeper training (QPR) for mentors; universal prevention focusing on stigma reduction; and identifying adolescents who have elevated signs of the most important risk factor for suicidal behaviors.	Gijzen et al. (2018)
Sources of Strength	The program recruits and trains key opinion leaders (i.e., peer leaders) along with school staff members as advisors.	Pickering et al. (2018, 2022)
Online psychoeducational program (ProHelp)	The program comprises two modules. Each module was designed to take approximately 5 min. The first module addresses suicide literacy, suicide and help-seeking stigma, and available help-seeking sources. The second module addresses self-reliance, social support, and myths about mental health professionals.	Han et al. (2018)
Mental Health First Aid program	The program was a 2.5-h course combining lectures, videos that demonstrated good and bad gatekeeper behavior, and small group role-plays along with the scenario of the videos.	Hashimoto et al. (2016)
Comprehensive suicide response program	The curriculum provided detailed lesson plans for three 40–45-min participatory classes.	Kalafat and Elias (1994)
Reframe-IT intervention	The intervention comprised eight modules based on cognitive behavioral therapy delivered online across the 10-week intervention period.	Robinson et al. (2014)
The Zuni Life Skills Development Program	Intervention strategies consistent with cultural and community life values and strengths.	LaFromboise and Lewis (2008)
Creating Suicide Safety in Schools (CSSS) workshop	A workshop was designed to encourage school personnel to evaluate their own schools’ existing suicide prevention and intervention readiness and to plan ahead.	Breux and Boccio (2019)
Jason Foundation (JF) “A Promise for Tomorrow” program	The program promotes awareness of the problem of youth suicide, provides student trainees with the knowledge and resources to interact with at-risk youth, and encourages referral behaviors.	Totura et al. (2019)
Applied Suicide Intervention Skills Training (ASIST)	The program is a 14-h, 2-day suicide intervention training mode. SafeTALK is a condensed version of ASIST.	Shannonhouse et al. (2017)
Thai Suicide Prevention Program for Secondary School Students (TSPPSSS)	The program comprised three modules targeting adolescent peer leaders, parents, and schoolteachers.	Chaniang et al. (2019)
Beyond the Wall	Death education program aimed at helping young people cope with being told of the suicide of a student at their school and to raise awareness of their negative emotions and their representations of death to improve their ability to cope with negative thoughts.	Testoni et al. (2020)
Safety Planning Intervention (SPI)	A structured personalized safety plan collaboratively completed by clinicians and clients to assist individuals in managing a suicidal crisis.	Stewart et al. (2020)
Student Assistance Program (SAP)	Team members identify student psychosocial problems, determine whether they are within the school’s realm of responsibility, and suggest interventions. When a problem is beyond the array of services provided at the school, teams assist in accessing services within the community.	Biddle et al. (2014)
“Talk-to-Me” Mass Open Online Course (MOOC)	Online psychoeducational suicide prevention program targeting young adults. This skills training program aims to increase young adults’ awareness of mental health-promoting activities, improve their resilience, develop their distress management skills and ability, and to identify the early signs of suicide ideation or behavior in themselves and others and apply suicide crisis intervention strategies.	Afsharnejad et al. (2022)
Hope Squad	Hope Squad is a school-based, peer-to-peer suicide prevention program across the United States in more than 1,000 schools across 33 states; the program has greater than 30,000 student members (see https://hopesquad.com/).	Wright-Berryman et al. (2022)
Online peer gatekeeper training program	The program covers mental health basics, current status of suicide problems, danger sign features of suicide, how to appropriately respond, demo video, and referral information for appropriate resources. Each section takes 10–20 min to view and contains a voiceover, cases, personal work, and quizzes.	Nozawa et al. (2022)
Multimodal suicide prevention program for young people	The program involves delivering universal psychoeducation (safeTALK) to all students, screening them for suicide risk, and delivering internet-based Cognitive Behavioral Therapy (Reframe IT) to those students identified as being at high risk for suicide.	Byrne et al. (2022)
Broad-Minded Affective Coping (BMAC)	A brief psychological intervention targeting suicidal ideation by enabling access to competing positive emotions and thoughts using guided imagery.	Knagg et al. (2022)
	*Intervention*	
School crisis intervention program	A crisis protocol made up of different phases to address suicide crisis management.	Cha et al. (2018)
Critical Incident Stress Management (CISM)	The program is a multicomponent, seven-step process that is based on step-by-step interventions, timing, activation, goals, and format.	Mintz-Binder (2007)
Consultation and Resource Evaluation (CARE) program	Essential components of the program include assessment of student suicide risk, evaluation of a student’s willingness and ability to refrain from self-harm; consultation regarding needed psychiatric, psychological, and supportive educational services; and parent information and supportive educational intervention.	Rivero et al. (2014)
	*Postvention*	
Suicide Postvention Guidelines for schools	Designed to help secondary schools develop an Emergency Response Plan (ER Plan) and Emergency Response Team (ER Team) following a student suicide within the school.	Cox et al. (2016)
Counselors, Administrators, Parents, and Teachers (CAPT) team approach	The approach could be adapted to include the prevention and intervention phases of dealing with teen suicides.	Maples et al. (2005)

**Table 3:** Educational setting of the studies.

Educational setting							
Phase	Primary school		Secondary and high school		College		
	*N* (%)	Studies	*N* (%)	Studies	*N* (%)	Studies	Total *N* (%)
Prevention	1 (1.51%)	Sattem (1990)	37 (56.06%)	Ahern et al. (2018); Bockhoff et al. (2022); Breux and Boccio (2019); Brown et al. (2018); Brown and Grumet (2009); Byrne et al. (2022); Chaniang et al. (2019); Chaniang et al. (2022); Fendrich et al. (2000); Flynn et al. (2016); Gijzen et al. (2018); Johnson and Parsons (2012); Kahn et al. (2020); Kalafat and Elias (1994); Knagg et al. (2022); Kinchin et al. (2020); LaFromboise and Lewis (2008); Marbaniang et al. (2022); Mascayano et al. (2018); McCalman et al. (2016); Pickering et al. (2018); Pickering et al. (2022); Roberts et al. (2018); Robinson et al. (2014); Ryerson (1990); Schilling et al. (2014); Schmidt et al. (2015); Shannonhouse et al. (2017); Stein et al. (2010); Testoni et al. (2018); Testoni et al. (2020); Tompkins et al. (2010); Totura et al. (2019); Wasserman et al. (2015); White and Morris (2010); Wright-Berryman et al. (2022); Zenere and Lazarus (2009)	21 (31.81%)	Muehlenkamp et al. (2009); Thompson et al. (2010); Indelicato et al. (2011); Fernández Rodríguez and Huertas (2013); Muriungi and Ndetei (2013); Yousuf et al. (2013); Cimini et al. (2014); Downs et al. (2014); Hashimoto et al. (2016); Pullen et al. (2016); Han et al. (2018); Coleman et al. (2019); Cramer et al. (2019); Chugani et al. (2020); Fernandes et al. (2020); Stewart et al. (2020); Willson et al. (2020); Afsharnejad et al. (2022); Cross et al. (2022); Nozawa et al. (2022); Pothireddy et al. (2022)	59 (89.39%)
Intervention	-	-	1 (1.51%)	Biddle et al. (2014)	2 (3.03%)	Mintz-Binder (2007); Rivero et al. (2014)	3 (4.54%)
Postvention	-	-	4 (6.06%)	Callahan (1996); Maples et al. (2005); Cox et al. (2016); Cha et al. (2018)	-	-	4 (6.06%)
	**1 (1.51%)**		42**(63.63%)**		**23 (34.84%)**		**66 (100%)**

Regarding geographical distribution, studies proceed from the United States (37), Australia (9), Germany (6), Italy (5), Austria, Ireland and Sweden (4), Canada (3), England (3), Estonia (3), France (3), Hungary (3), Israel (3), Romania (3), Eslovenia (3), Spain (3), Japan (2), China (2), Switzerland (2), Chile (1), India (1), Kenya (1), Netherlands (1), Norway (1), South Korea (1), and Thailand (1). Notably, there are studies that proceed from more than one country. Wasserman et al. (2015), Kahn et al. (2020), and Ahern et al. (2018) analyzed data from 10 European countries: Austria, Estonia, France, Germany, Hungary, Ireland, Italy, Romania, Slovenia, and Spain. Han et al. (2018) analyzed data from China and Australia. Cox et al. (2016) analyzed data from Australia, New Zealand, and the United States.

### 3.1. Agents called into action in the educational context

The literature identifies various agents that play a role in suicide prevention, intervention, and postvention. Tierney et al. (1990) contend that every major stakeholder group in the school system, including board members, administrators, professional staff, support staff, parents, and students should participate (Tierney et al., 1990). Similarly, other authors have called for the whole school community to take responsibility for addressing suicide in educational contexts (Ryerson, 1990; Maples et al., 2005; Tompkins et al., 2010; Cox et al., 2016; Shannonhouse et al., 2017; Gijzen et al., 2018; Roberts et al., 2018).

In the primary school context, Roberts et al. (2018) identified agents such as teachers, psychologists, counselors, and parents. In secondary and high school contexts, the literature identified a wide range of agents who should intervene in the fight against suicide: teachers, school guidance counselors, school nurses, parents, school-based mental health professionals, such as school counselors, social workers, and school psychologists and adolescents themselves. Various agents were identified as having a role in suicide prevention, intervention, and postvention initiatives in college: college students, student organization representatives, administration staff living on campus, student affairs staff and administrators, parents and family members, college counselors, college psychologists, college faculty and staff and trained facilitators, clinical professionals who can evaluate mental health problems, campus ministers, university police officers, public safety and transportation personnel, and tribal leadership in the case of suicide attempts in American Indian communities.

### 3.2. Programs’ characteristics and outcomes

Overall, the programs were described as safe (Robinson et al., 2015), contributing to school safety (Breux and Boccio, 2019), and feasible to implement within a school setting (Kinchin et al., 2020). Educational initiatives addressing suicide had a positive impact on participants’ levels of knowledge, attitudes, and beliefs regarding suicide and suicide prevention (Kalafat and Elias, 1994; Tompkins et al., 2010; Indelicato et al., 2011; Yousuf et al., 2013; Schilling et al., 2014; Schmidt et al., 2015; Flynn et al., 2016; Roberts et al., 2018; Chaniang et al., 2019; Coleman et al., 2019; Cramer et al., 2019; Totura et al., 2019). Testoni et al. (2020) reported that participants who received education about death showed improvements in the positive meaning of life and reduced anxiety. Additionally, they identified improvements in students’ ability to recognize emotions and communicate them verbally (Testoni et al., 2020). Ryerson (1990) reported an increase in the number of referrals to the local mental health provider, less resistance to asking for help, improved communication, enhanced trust between students and suicide prevention program personnel, and a decrease in the number of suicides in participating school systems (Ryerson, 1990). Zenere and Lazarus (2009) and Wasserman et al. (2015) found that comprehensive school-based suicide prevention programs reduced youth suicidal behavior. Conforti et al. (2020) showed that a teacher-delivered cognitive behavior therapy skills curriculum was feasible and associated with reduced suicidality (ideation and behavior) in middle school-aged youth.

Breux and Boccio (2019) provided preliminary evidence on the effectiveness of suicide educational programs. The programs improved participants’ attitudes toward the importance of school-based suicide prevention, understanding of best practices, perceptions of administrative support, and feelings of empowerment to work collaboratively and enhance their school’s suicide safety. Educational stakeholders who received training in suicide prevention reported feeling more comfortable, competent, and confident in intervening with a person at risk of suicide (Muehlenkamp et al., 2009; Johnson and Parsons, 2012; Cimini et al., 2014; Hashimoto et al., 2016; Shannonhouse et al., 2017; Brown et al., 2018; Stewart et al., 2020).

Some negative outcomes of educational suicide interventions were also reported in the literature. Fendrich et al. (2000) showed that the unsolicited mass distribution of information and materials related to suicide and violence prevention is of limited usefulness. Maples et al. (2005) described the corrections made to a suicide crisis management intervention to avoid romanticizing suicide. Callahan (1996) described how a sense of “specialness” and secrecy served to heighten students’ sense of melodrama over a school mate’s suicide, which also furthered the spread of suicide. However, when he altered the postvention activities to avoid the atmosphere of romantic tragedy, such as reporting every expression of student suicide ideation to parents regardless of the level of severity, suicidal ideation decreased. In fact, this communication with parents was helpful because it focused attention on parent–child conflicts, thus making it possible to solve family issues that, in some cases, were contributing to suicidal ideation. Roberts et al. (2018) pointed out the importance of offering primary school teachers coaching and support in addition to regular training for addressing suicide. An in-depth qualitative study by White and Morris (2010) showed that teachers might feel insecure about approaching the subject of suicide with students and use fact-based information without giving students the opportunity to conceptualize suicide as a social historical phenomenon. White and Morris (2010) warned that there might be unexpected and sometimes unwanted learning during suicide educational initiatives. Breux and Boccio (2019) cautioned that insufficient time and stigma surrounding the topic of suicide are barriers to implementing changes after educational interventions. Han et al. (2018) reported that the program had a short-term positive influence on participants’ suicide literacy, although it was not sufficient to change students’ attitudes or intention to seek help. Finally, the effects of gatekeeper suicide prevention training over time have been found to be unsustainable in studies that incorporated a follow-up step in their methodology (Cimini et al., 2014; Brown et al., 2018).

### 3.3. Recommendations

The literature presents numerous recommendations based on the implementation and assessment of educational interventions for suicide. As 59 of the 66 articles included in this review address suicide prevention, these recommendations globally apply to the prevention setting. However, we would highlight, in line with Mintz-Binder (2007) who presents a study addressing suicide intervention, that if a suicide occurs, faculty and staff involved in teaching should neither be expected to handle these events alone nor be made to feel guilty. Mintz-Binder (2007) urges educational institutions to have a well-rehearsed plan established before these sudden events occur. This can help minimize the shock and denial responses in this traumatic situation, enabling an organized, systematic approach to be implemented.

Turning now to preventive recommendations, Willson et al. (2020) pointed out the need to continue addressing biases and stigma surrounding suicide. Tompkins et al. (2010) advised educational communities to come together to talk about suicide prevention, identify weaknesses, build on strengths, and create plans of action. Afsharnejad et al. (2022) encourage suicide prevention interventions among tertiary students to consider using online peer mentoring programs to create user groups where participants can practice their skills face-to-face.

Wasserman et al. (2015) stressed a need for the large-scale implementation of universal school-based suicide prevention programs. Ryerson (1990) recommended that extensive research into the target educational context and student population should be conducted before initiating a suicide educational program and that as many key players as possible should be involved in the tailoring process. Tierney et al. (1990) stated that a suicide prevention program must be based on a system-wide policy and address all aspects of suicide: prevention, intervention, and postvention. Tierney et al. (1990) recommended the creation of comprehensive programs that require coordination and networking components, along with implementation commitments from every major stakeholder group in the school system. These included board members, administrators, professional staff, support staff, parents, and students.

Shannonhouse et al. (2017) stated that training is needed in school settings to respond to young people at risk of suicide. School counselors should be trained in suicide intervention skills to build the capacity of their school community and provide suicide first aid to students in need. Cox et al. (2016) recommended that school staff should not use the terms “committed suicide” or “successful suicide” when discussing a death because the word “committed” is associated with an illegal or criminal act, and “successful” implies that the individual achieved a desirable outcome. Johnson and Parsons (2012) and Shannonhouse et al. (2017) recommended that suicide should be a training priority for school staff. Every front-line staff member should know how to intervene with potentially lifesaving responses (Johnson and Parsons, 2012). Similarly, Brown et al. (2018) recommended gatekeeper workshops as school staff are important gatekeepers in preventing adolescent suicide.

However, Roberts et al. (2018) warned that teacher training alone is insufficient to ensure that teachers impart mental health promotion strategies to their pupils. They argued that teachers also need ongoing support and coaching throughout the school year if their students are to learn and integrate mental health strategies. With appropriate guidance and support, schools can be integrated into the tapestry of social institutions working to reduce the loss of young life to a preventable public health problem (Breux and Boccio, 2019).

Additionally, isolated training sessions are not recommended. Various studies highlight the value of periodic suicide prevention training and exposure to a variety of models to provide or reinforce corrective educational and practical experience (Kalafat and Elias, 1994; King and Smith, 2000; LaFromboise and Lewis, 2008; Indelicato et al., 2011). Johnson and Parsons (2012) recommended updating knowledge and skills training to mitigate erosion in confidence and increase the likelihood of effective intervention. Cimini et al. (2014) recommended booster training sessions to address skill degradation over time.

Stein et al. (2010) suggested that suicide prevention training should educate school personnel about the key components of guideline-based suicide prevention services, including information about confidentiality. The training should also suggest alternative strategies to respond to unique educational context needs, populations, and institutional resources. Roberts et al. (2018) additionally suggested that each audience member should take a pretest prior to each suicide prevention educational session to assess pre-existing knowledge levels.

Schmidt et al. (2015) proposed that educational suicide prevention efforts in schools should also focus on issues such as family problems, grief or loss, and being bullied as factors associated with suicidal thoughts. Biddle et al. (2014) further suggested psychological autopsies for all adolescents who died by suicide. Pickering et al. (2018) recommended peer-led interventions as an important complement to other intervention strategies targeting higher-risk youth. According to Cimini et al. (2014), implementing audience-specific gatekeeper training programs can be beneficial. Brown and Grumet (2009) contended that when considering screening for mental health issues in schools, the ability to follow up with at-risk youth is essential. They further stated that it is essential for positively screened young people to be linked to some additional evaluation or treatment and that this should not be decided solely by the parents. Cha et al. (2018) warned that having a crisis protocol intervention when a peer suicide occurs helps to improve trauma-related symptoms and might be an effective way to prevent suicide from spreading among students by alleviating such trauma-related symptoms.

Additionally, White and Morris (2010) highlighted the complexity of suicide as a culturally situated phenomenon. They argued against conceptualizing suicide through singular, stable, or universalizing terms that transcend time and context. They also claim that several factors contradict the overall aims of youth suicide prevention. These include expecting educators to rely exclusively on narrow “evidence-based” curricula that authorize expert knowledge and preclude all other knowledge, identifying problems within people, dismissing any uncertainty or ambiguity, inhibiting local and relational meaning-making, and stifling creativity by rigidly adhering to pre-specified and “safe” learning outcomes.

Regarding recommendations made specifically for young adults, Fernandes et al. (2020) discussed the importance of developing projects for the university community. Given the need to discuss and reflect on suicide prevention, they recommend that these projects be integrated with the health network and student support services of educational institutions. Chugani et al. (2020) recommended that campuses that can invest additional resources in student mental health and suicidality should focus on primary prevention, such as increasing coping skills and resilience. Rivero et al. (2014) suggested that campus staff should consider the array of policies, programmatic infrastructure, on- and off-campus mental health, and other support resources that can be mobilized so that each student can be managed according to their needs.

The literature review also identified recommendations regarding the dissemination of materials related to suicide. Fendrich et al. (2000) warned that when unsolicited materials are sent to schools, the most appropriate school contact person should be identified in advance. Their experience shows that distribution to the right contact person, especially when preceded by personal contact through telephone calls, is more likely to result in effective dissemination than a mass-mailing approach. Indelicato et al. (2011) and Han et al. (2018) also recommended that future suicide prevention intervention programs for university students should consider an online approach, as students generally favor that mode.

Finally, recommendations have been made regarding interventions within tribal communities (LaFromboise and Lewis, 2008). Lafromboise and Lewis (2008) strongly recommended that these interventions include protocols associated with cultural resources, indigenous values, and healing practices. They suggested that researchers should seek guidance from tribal/community leaders to develop and apply such interventions. If interventions are to be conducted effectively, researchers must intervene in the most professional and culturally competent manner possible (LaFromboise and Lewis, 2008).

## 4. Discussion

A high percentage of the studies included in this review used quantitative methodology to reach their objectives (75.75%), which is helpful for objectively assessing the viability and effectiveness of the different programs. However, more qualitative or mixed studies are also needed to analyze aspects that quantitative procedures cannot assess or identify. Regarding the geographical distribution of the studies, the vast majority were carried on in the United States. This result coincides with other systematic reviews in other contexts; thus, considerably more studies are needed in other countries and cultural settings. According to the World Health Organization (2021a, 2021b), suicide rates vary considerably among countries, which suggests that sociocultural variables may explain suicidal behavior to some extent. Goldston et al. (2008) argue that consideration should be given to cultural patterns related to suicide, such as the kind of triggers or precipitants of suicidal behavior, the reactions to and interpretations of suicidal behaviors, and the search for help, which may vary across cultures. Furthermore, risk and protective factors for suicidal behavior may also be influenced by cultural context (Goldston et al., 2008). For this reason, research efforts should prioritize interventions in diverse cultural contexts and countries, as certain programs may be more suitable for specific settings. It is equally important to develop programs tailored to cultural characteristics and rigorously assess their effectiveness. The lack of culturally sensitive prevention programs tailored to educational contexts is a significant limitation that could result in economic and human costs. Hence, it is imperative to address this gap to create more effective and inclusive suicide prevention strategies.

Most studies focus specifically on suicide prevention, particularly in secondary and high schools. The focus on intervention and postvention efforts in the aftermath of suicide acts is less prominent. Therefore, more studies are needed on the development and assessment of intervention and postvention programs in the educational context. In fact, Tierney et al. (1990) pointed out that programs to reduce suicidal behavior should address all aspects of suicide, including prevention, intervention, and postvention. Furthermore, one positive outcome of the current review is that it has identified a wide range of stakeholders at different educational levels, including students, teachers, counselors, families, psychologists, administrators, and staff. However, some programs are not designed for the entire educational community, a limitation that several authors point out that needs to be redressed (Ryerson, 1990; Maples et al., 2005; Tompkins et al., 2010; Shannonhouse et al., 2017; Berk and Adrian, 2018; Gijzen et al., 2018). This can be achieved by developing comprehensive programs that facilitate the commitment of different stakeholders and the coordination between them (Tierney et al., 1990). One notable positive aspect arising from the focus on stakeholders at various educational levels in the present study is the potential for a more comprehensive and holistic approach to suicide prevention within educational institutions. This inclusive approach allows for a broader perspective on addressing the issue. It facilitates the identification of key individuals and groups that can play a significant role in shaping effective prevention, intervention, and postvention strategies. It underscores the need for more targeted and cohesive initiatives that ensure stakeholders’ active involvement and coordination, as highlighted by Tierney et al. (1990).

Most suicide educational programs are effective in terms of changing students’ understanding, knowledge, perceptions, and attitudes (Kalafat and Elias, 1994; Tompkins et al., 2010; Chaniang et al., 2019; Coleman et al., 2019; Totura et al., 2019). More specifically, those who attended suicide educational interventions were reportedly more knowledgeable about suicide prevention after the educational sessions and had more helpful attitudes or beliefs about suicide. However, Han et al. (2018) suggested that improved understanding in the short term does not necessarily change the intention to seek help when experiencing suicidal ideation, which may limit the real impact of programs that only assess changes in students’ understanding. The work by Zenere and Lazarus (2009), Wasserman et al. (2015), and Conforti et al. (2020) suggested that suicidal ideation and behavior were reduced. Of the 58 studies analyzed, only four reported attendees actually practicing their new abilities. Johnson and Parsons (2012) reported that within 3 months of training, one staff member reported using the Question, Persuade, and Refer (QPR) response with a suicidal student. Stewart et al. (2020) stated that two-thirds of the clinical staff who attended training implemented suicide prevention initiatives at least once. Coleman et al. (2019) reported a medium-sized increase in the number of peers referred to mental health services by participants in an educational suicide initiative. Hashimoto et al. (2016) mentioned that one-third of participants had one or more opportunities to use their suicidal student management skills within a month. None of these cases assessed how attendees of educational suicide initiatives had changed their practice using their new suicidal student management skills.

Much more evidence is needed on the long-term impact of prevention, intervention, and postvention programs and whether they lead to deeper changes in students, which effectively reduces suicidal behavior in the long term. Moreover, according to Roberts et al. (2018), the assessment of prevention programs should include a comparison between a pre-test before the implementation of the program and a post-test after the program to determine whether there have been any changes. Evaluating suicide educational programs reveals a positive outcome, showcasing their effectiveness in bolstering students’ knowledge and fostering more constructive attitudes toward suicide prevention. Nonetheless, a critical examination of the findings underscores crucial areas for enhancement. While short-term understanding is essential, it must be accompanied by a tangible intention to seek help, a fact that some studies suggest might be lacking. Moreover, the application of acquired skills within the educational community remains unexplored, leading to a gap in understanding how program attendees translate knowledge into practical changes when addressing suicidal students.

Several recommendations were made by the authors of these studies. These included the need for school staff and counselors to be trained (Johnson and Parsons, 2012; Shannonhouse et al., 2017); addressing biases and stigma about suicide (Willson et al., 2020); providing guidance, support, and coaching to teachers on mental health strategies (Roberts et al., 2018); and implementing prevention programs periodically to increase their impact (Kalafat and Elias, 1994; King and Smith, 2000; LaFromboise and Lewis, 2008; Indelicato et al., 2011). Studies also highlighted the need for these programs to address issues that may have a negative impact on the mental health of students, such as bullying and family problems (Schmidt et al., 2015), the need to follow up with at-risk students (Brown and Grumet, 2009), and the need for educational institutions to have a crisis protocol intervention to minimize negative reactions to a peer suicide or a sudden event (Mintz-Binder, 2007; Cha et al., 2018).

In conclusion, the current systematic review identifies educational agents and institutions called into action in suicide prevention. It provides an overview of the prevention, intervention, and postvention programs carried out in educational institutions to reduce suicidal manifestations and shows the state of current practice. The study describes the different types of programs that have been provided, the countries in which they have been implemented, and the agents who have been targeted as well as the recommendations given by various authors. It also identifies gaps in the research on suicide in education, such as the need (1) for more qualitative or mixed studies that assess or identify aspects that are not easily explored with quantitative procedures, (2) to diversify the countries and cultural contexts in which educational initiatives on suicide are carried out, (3) to promote interventions and postventions in the aftermath of suicide acts, and, most importantly, and (4) to reduce suicidal ideation and behavior by doing more than simply identifying participants’ perception of changes in their understanding of and attitudes toward suicide and suicide prevention. This information may be helpful in designing and developing appropriate new research projects and programs for reducing suicidal behaviors in educational settings.

This study has some limitations that must be considered when interpreting its results. First, it is essential to note that most of the research in the review comes from the United States, with 33 articles out of 66 used; this could imply that the findings more accurately reflect the country’s reality. Second, although systematic reviews are a rigorous research methodology, it is essential to recognize that they do not allow statistical analysis of results drawn directly from primary studies, as meta-analyses do. This difference in methodological approach could have implications for interpreting the results and their generalization to other contexts.

On the other hand, it is necessary to consider the possible publication bias in the scientific literature. It is common for research with negative results to be less likely to be published, which could lead to overestimating the real effect of educational interventions on suicide prevention. It is essential to encourage the publication of all positive and negative results to obtain a more complete and accurate picture of the effectiveness of interventions in this field.

## Author contributions

JMDO, J-MD, and FM-V contributed to conception and design of the study, performed data extraction and screening and quality analysis, and wrote sections of the manuscript. EG-N organized the database. JMDO wrote the first draft of the manuscript. All authors contributed to the article and approved the submitted version.

## Funding

This work is part of the PECT “Pobles Vius i Actius” project, Operation “Formació i Tecnologia”, within the frame of the RIS3CAT and ERDF Catalonia Operational Programme 2014–2020. It is co-financed by the Catalan Government and the Provincial Council of Tarragona.

## Conflict of interest

The authors declare that the research was conducted in the absence of any commercial or financial relationships that could be construed as a potential conflict of interest.

## Publisher’s note

All claims expressed in this article are solely those of the authors and do not necessarily represent those of their affiliated organizations, or those of the publisher, the editors and the reviewers. Any product that may be evaluated in this article, or claim that may be made by its manufacturer, is not guaranteed or endorsed by the publisher.
